# Preparedness of County Referral Health Facilities in Implementing Adolescent Friendly Health Services: A Case Study of Mama Lucy Kibaki Hosptal

**DOI:** 10.5539/gjhs.v7n6p11

**Published:** 2015-03-25

**Authors:** Pacific Akinyi Owuondo, Wanja Mwaura-Tenembergen, Maureen Adoyo, Elizabeth M. Kiilu

**Affiliations:** 1 Mama Lucy Kibaki County Referral Hospital, Kenya; 2 Department of Health System Management, Kenya Methodist University, Kenya

**Keywords:** preparedness, staff capacity, health resources, adolescent friendly health services

## Abstract

Health service delivery is a key pillar of the health system management .The World Health Organization recently emphasized the need to develop adolescent -friendly health services to improve the care provided to young people throughout the world. However, there is limited peer reviewed literature on this subject therefore necessitating assessment of whether the existing health facilities are prepared to implement the adolescent friendly health services. Adolescent friendly health services remains a relatively new and sensitive area mainly due to restrictive norms and policies guiding the services. After International Conference on Population and Development in 1994, countries started implementing adolescent friendly health services. The Government of Kenya together with partners in an attempt to address the health challenges came up with the Adolescent package of care (APOC) in 2013 whose guidelines were finalized in November 2014 and released for use by service providers . Despite this package of care, there is still ineffective staff capacity in relation to skills and knowledge gap of health professionals, training needs, health resources as well as health system factors that can affect implementation of AFHS. The study explored ways of mitigating or addressing the barriers to implementation of these services. The study used both quantitative and qualitative approaches to collect data. The study utilized survey research adapting descriptive cross sectional design and semi-structured questionnaire to interview 348 health care providers and 472 adolescents in Mam Lucy Kibaki Hospital from 3rd May 2014 to 16 June 2014 .The key informants were mainly nurses, clinical officers and Medical doctors who were working at the health service delivery area at the time of study and were interviewed using an interview guide. The managers at the hospital were interviewed using an in-depth interview guide while the adolescents were interviewed through interview guide and focused group discussion. Quantitative data was analyzed using Statistical Package for Social Sciences (SPSS) Version 18.0. Descriptive statistics and chi-square tests were performed to determine significant associations. The study established that sex, age, level of education and adolescent awareness about existence of friendly health services offered were significantly associated with utilization at p<0.05. Long queues, unfavorable working hours and lack of money negatively affected consumption of AFHS.The study concluded that the utilization of health services among the adolescents was low largely due to unfriendliness of the health care providers at health facilities and lack of awareness of AFHS services. In view of the findings, this study recommends need for the Government through the Ministry of Health and partners in health service provision to increase the number of AFHS and ensure that the recommendations of Adolescent Package of care is implemented fully with good evaluation strategies in place. Laborious awareness drives to sensitize the adolescents about the available services through rigorous health education and increased involvement of both parents/guardians and teachers to scale up implementation are also recommended.

## 1. Introduction

### 1.1 Background Information

Health systems have been defined in various ways. The most widely-used definition is from the World Health Report 2000, which defines health systems functionally as “all the activities whose primary purpose is to promote, restore or maintain health.” (WHO, 2000). These activities are often grouped into six pillars or “building blocks”, namely 1) service delivery, 2) health workforce, 3) health information systems, 4) medical products, vaccines and technologies, 5) health systems financing and 6) leadership and governance (WHO, 2007). Health systems has also been defined as all the activities whose primary purpose is to promote, restore and/or maintain health (WHO, 2010), the people, institutions and resources, arranged together in accordance with established policies, to improve the health of the population they serve, while responding to people’s legitimate expectations and protecting them against the cost of ill-health through a variety of activities whose primary intent is to improve health (WHO, 2012).

Health service delivery is a key pillar of the health system management. The World Health Organization (WHO, 2000) defines *service delivery* as the way inputs are combined to allow the delivery of a series of interventions or health actions (WHO 2001b). Service delivery is the chief function of a health system (WHO, 2003). It further acknowledges that a strong, well-functioning and sustainable health system is that which has the capacity to efficiently deliver and maintain health care services by ensuring adequate staff capacity, health system and health resources (WHO, 2002).

Globally, there are 1.7 billion young people aged 10 to 24 years, representing one- quarter of the world’s population, with over 85% living in developing countries (Population Reference Bureau, 2013). In Kenya, statistics from Kenya National Bureau of Statistics (KNBS) census report estimate the youth to be about 40% of the population and youth aged 10-24 years make up to 36% of the population (KNBS, 2010). Additionally, 30% of Kenyan population is in the adolescent age group of 10-19 years (KNBS, 2010). They further estimated that there are almost 12 million adolescents in Kenya. They represent a resource for the future whose potential can either be wasted or nurtured in a positive manner. However, access to health care services by adolescents has been a challenge globally and especially in developing countries.

WHO (2009) identified and recommended that, Adolescent, health issues are addressed through the promotion of responsible and healthy behavior, including the provision of appropriate services and counselling specifically suitable for that age group (WHO, 2002a). Countries were encouraged to ensure that programmes and attitudes of health-care providers do not restrict adolescents’ access to the services and information they need. These services must safeguard the right of adolescents to privacy, confidentiality, respect and informed consent, while respecting cultural values and religious beliefs as well as the rights, duties and responsibilities of parents (WHO, 2007).

Ministry of Health in Kenya formally approved the country’s first Adolescent Package of Care (APOC) to sensitize health service providers on the adolescent package of care in Kenya and enhance knowledge and skills in delivery of adolescent care. It focuses on: Stages of adolescent development, clinical assessment, mental Health, communication and counseling, nutritional care and support, psychosocial support sexual and reproductive health, community level care and support, adolescent/youth friendly services transition of Adolescent Living with Human Immuno-Deficiency Virus (PLHIV) from pediatric to adult care .

Despite these initiative, adolescent friendly health service implementation still faces a lot of challenges related to the sensitive nature of adolescent health and poor health system structures hence under implementation (APOC, 2014). The current health care services given to adolescents mainly focus on services such as physical environment and sanitation, nutritional status, immunization and treatment of common illnesses. It is because of this that there is need to assess the preparedness of Mama Lucy Kibaki Hospital to deliver friendly health services to adolescents such as sexual and reproductive health, mental health ,life skills as well as nutrition services among others . Furthermore it is crucial for all health care providers offering services to adolescents to have the right knowledge, skills, training, attitudes and values to undertake health care service delivery.

Consequently, the current constitution requires that health service delivery is devolved at county level which further adds to the government commitment on delivering health services to its citizens. Therefore, in order to build staff capacity and health resources at county level, health workers require new capacity building and technical support to deliver AFHS at through on job training (MOH, 2012). According to Kipchumba (2012) effective delivery of health care services at county level can only be achieved if the government invests in training health care workers on how to adjust from delivering health care services at national level to county level.

### 1.2 Brief History of Mama Lucy Kibaki Hospital

Mama Lucy Kibaki Hospital is a government county referral hospital serving the residents of Nairobi’s populous Eastland. The institution is a key development which was established in 2011 but officially opened in 2013. The hospital has expanded health services in Kenya, not only to people in the Eastern part of Nairobi County in Kenya, but also to all Kenyans from all over. Mama Lucy Kibaki Hospital in Nairobi has also reduced the pressure on the Kenyatta National Hospital in Upper Hill part of Nairobi which was previously serving the Nairobi area residents. The hospital has a bed capacity of 112 offering specialized services for both outpatient and in-patient cases. Also available are laboratory, maternity and minor surgeries. It is the Government’s efforts to strengthen the health system by providing comprehensive health services to all Kenyans.

### 1.3 Statement of the Problem

As a response to the health needs of adolescents, the Ministry of Health initiated integration process of priority concerns into the Kenya Essential Package for Health (KEPH) Programme at especially the community level of health care. The government further adopted the Adolescent Package of Care (APOC) in 2014 with a commitment to address adolescent health issues raised by the National AIDS/STI Control Program. The goal of this APOC was to increase the proportion of facilities offering AFHS to 90%, up from 7% as at that time and reduction of the proportion of women aged below 20 with a first birth from 45% in 1998 to 22% (KNBS, 2010) .This was far below expectation in meeting the health needs of the 30% adolescent population in Kenya (KNBS, 2010). Adolescent have numerous health concerns that need to be addressed at least in part through the health care system. Moreover, very few programs have been able to implement the special health needs of adolescents. In addition, there is less focus on adolescent by these programs and policies.

The significant features of an Adolescent Friendly Health Center/Clinic (AFHC) encompass provision of comprehensive health services to adolescents (AFHC, 2008). The behavioral patterns established during the second decade of life help influence not only the current health status of adolescents, but their risk for developing chronic diseases into adulthood (APOC, 2014). However most of county health care facilities in Kenya are lacking systems in place to address these concerns such as sufficient health care amenities to deliver AFHS. Thus questioning their preparedness in implementing adolescent friendly health services (MOH, 2012). Braeken (2004) asserts that staff capacity and health resources are vital for the implementation of AFHS and therefore the obvious need to build on existing health professional’s capacity.

According to International Planned Parenthood Federation (IPPF, 2008) the overall goal of AFHS is to effect positive health among the adolescents. Adolescents continue to remain at risk because they pose different challenges for the health-care system than children and adults, due to their rapidly evolving physical, intellectual and emotional development. Thus calling for development and strengthening of need based interventions. It is these revelations that prompted this study.

## 2. Research Design

The report adopted a cross-sectional design. Orodho (2003) States that cross-sectional design is good because it provides a snapshot of the exposed variables (staff capacity and health resources) across a wide population without manipulating or influencing the study population in any way. Cross-section design was also used because it can produce multiple outcomes and various exposures/variables can be studied at a given point in time.

### 2.1 Target Population

The study was conducted among the managers, health care professionals and Adolescents who seek for health care services at Mama Lucy Kibaki Hospital. The study also targeted the 452 adolescents who visit the health facility every month. The distribution of the population is shown in Table 1.

**Table 1:** Target Population (Source: Author, 2014)

Category	Population N (%)
Managers	10 (1.4%)
Health care professionals	231 (33.3%)
Adolescents	452 (65.3%)
TOTAL	693 (100%)

### 2.2 Sampling Procedure

#### 2.2.1 Study Site

The study was carried out at Mama Lucy Kibaki hospital. The health facility is located within Eastern Part of Nairobi County. The location was appropriate as it provides monthly health care services to about 452 adolescents, hence was instrumental in providing appropriate information on the same.

#### 2.2.2 Inclusion Criteria

This comprised of all the managers, health care professionals working at Mama Lucy Kibaki Hospital at the time of study. It also included adolescents who visit the hospital for friendly health care services.

#### 2.2.3 Exclusion Criteria

The respondents who declined to give informed consent, those who are below 10 years or above 19 years and those who are not working at Mama Lucy Kibaki Hospital at the time of the study.

#### 2.2.4 Sampling Procedure Determination and Sampling Technique

2.2.4.1 Sample size Determination

The researcher used Yamane’s formula (1967) to arrive at the sample size for the health care professionals and adolescents. On the other hand census was used to sample managers. Calculation below illustrates how the sample size was arrived at.

2.2.4.2 Sampling Techniques

Stratified Sampling was used to select health care providers who offer services at Mama Lucy Kibaki Hospital showing different carders and the adolescents who seek health care services at MLKH selected from both in and out patient using simple random sampling. The adolescents were stratified as either male or female. On the other hand the managers were sampled using census.

### 2.3 Methods of Data Collection

1). Self-administered structured questionnaires to collect data from the health workers; 2). Self- administer questionnaire and an Interview guide for the managers. Finally four organized focused group discussion of 30-35 adolescents each with partial assistance from research assistants for adolescents who needed clarification. The group discussion was to determine feelings, perceptions and manner of thinking of adolescents regarding friendly health services in the hospital. All participants including focus group participants had to sign individual confidentiality forms in addition to consent/assent forms. Confidentiality was emphasized to all group members and why it was important for all involved to respect confidentiality. I found that providing a detailed explanation to participants and reasons for not disclosing what is discussed outside the focus group worked well. Data collection took one and half month since other health care providers were away on leave. On the other hand secondary Data were collected from government documents including Mama Lucy Kibaki Hospital health records journals and books. Information from these sources was used during literature review and for discussion.

### 2.4 Ethical Consideration

Permission to conduct the study was sought from the Scientific and Ethical Research Committee (SERC) of Kenya Methodist University, from Nairobi County Health Operational Research Technical Working group as well as from Training and Research Committee of Mama Lucy Kibaki Hospital. The researcher assured the respondents utmost approval by getting Informed consent from the study participants above 18 years. Adolescents who were under 18 years old were asked for their assent to be involved in the study. Confidentiality was maintained throughout the study. Respondents did not receive any incentives to participate in this study and no participant was forced to answer questions they did not wish to answer.

### 2.5 Data Analysis and Interpretation

Completed instruments were assembled, edited, coded and interpreted in relation to the research objectives. After entry, cross tabulation was done followed by chi square statistics to get the independent variables that were significantly associated with implementation of AFHS p< 0.05. After chi square, Odds ratio was done to check the direction of association and to test hypothesis. Qualitative data was transcribed and analyzed thematically and used in the discussion of results. Quantitative data was analyzed using SPSS Version 17.0 programme for data analysis. Demographic data was be presented and summarized using descriptive statistics, including frequencies, percentages and stratified by gender and age. Tables are used to summarize the frequencies of basic demographic and education characteristics of study participants included in the sample.

### 2.6 Expected Outcomes

The primary purpose of this research was to assess the preparedness of county referral health facilities in implementing AFHS. It has given insight to strengthen health service delivery to adolescents by providing evidenced based strategies on to improve staff capacity and resources at MLKH for adolescent health needs. This is intended to improve the health outcomes of the population and the necessary recommendations have been given to facilitate policy formulation, effective decision making, planning and action.

## 3. Results and Discussion

### 3.1 Descriptive Information of Study Variables

The study involved a total of 462 participants aged between 10-19 years, 231 key informants who were health care providers working at MLKH and 10 managers working at MLKH at the time of study. Table 2 below summarizes the descriptive information Adolescents.

**Table 2:** Demographic characteristics of Adolescents

Socio-Demographic Data		Respondent

Adolescents		N (%)
Gender	Female	156 (73.6%)
	Male	56 (26.4%)
Age (Years)	10 to 15	61 (28.8%)
	16 to 20	151 (71.2%)
Education Level	Primary	38 (20.8%)
	Secondary	68 (37.3%)
	College/University	76 (42%)
Relationship	In Relationship	82 (38.7%)
	Not in relationship	92 (43.4%)
	Married	38 (17.9%)

Table 2 shows that most of the adolescents interviewed, 156 (73.6%) were female while 56 (26.4%) were male. Out of a total of 212, 61 (28.8%) adolescents were within the age group of 11-15 years while 151 (71.2%) were within the age group 15-19 years. Also, majority of the adolescents interviewed, 181 (85.4%) were in school while 31 (14.6%) were not schooling. Of the 85.4 % in school, 38 (20.8%) were in primary schools, 68 (37.3%) in secondary and 76 (42.0%) were in college/university. 82 (38.7%) of the respondents were in an opposite sex relationship, 38 (17.9%) were married. Only 92 (43.4%) of the respondents were not involved in any opposite sex relationship indicating that the adolescents are in the process of transition from childhood to adulthood.

Table 3 shows that among the health care providers interviewed, majority 101 (69.2%) were nursing officers. The others were 18 (12.4%) clinical officers, 14 (9.6%) medical officers, 3 (2.1%) radiographers, 2 (1.4%) social workers, 7 (4.8%) records clerks/officers and 1 (0.7%) pharmacist.

**Table 3:** Demographic characteristics of Health Care Providers

Socio-Demographic Data of Healthcare providers		
Gender	Female	50 (34.2%)
	Male	96 (65.8%)

Age (Years)	< 20 years	1 (0.7%)
	21-25 years	16 (11%)
	25-35 years	69 (47.3%)
	35-45 years	47(32.2%)
	45-55 years	11(7.5%)
	55 + years	2 (1.4%)

Education Level	Certificate	4 (2.7%)
	Diploma	93 (63.7%)
	Higher Diploma	28 (19.2%)
	Bachelor Degree	19 (13.0%)
	Post graduate Degree	2 (1.4%)

Service Duration	< 1 year	34 (23.3%)
	1-2 years	66 (45.2%)
	2-3 years	41 (28.1%)
	3-5 years	5 (3.4%)

Job Designation	Nursing Officers	101 (69.2%)
	Clinical Officers	18 (12.4%)
	Medical Officers	14 (9.6%)
	Other carders	13 (8.8%)

More than half 96 (65.8%) health care providers interviewed were female; while male were 50 (34.2%). Of the health care providers interviewed,16 (11%) were aged 21-25 years, 69 (47.3%) aged 25-35 years, 47 (32.2%) were under 35-45 age group, 11 (7.5%) were in the age group 45-55 and 2 (1.4%) were officers aged above 55 ,only one officer was under the age of 20 years .

Majority, 93 (63.7) of the healthcare providers were diploma holders, higher diploma holders were 28 (19.2%), bachelor degree holders 19 (13.0%), certificate holders were 4 (2.7%) while postgraduate holders were only 2 (1.4%).

More than three quarters 119 (81.5%) of health care providers interviewed were married, 25 (17.1%) were single and 2 (1.4%) were widowed. However, most of the respondents 66 (45.2%) had 1-2 years of experience, 41 (28.1%) had 2-3 years of experience, 34 (23.3%) had worked in the hospital for less than a year and 5 (3.4%) had been offering healthcare services for a period 3-5 years.

The facility has 101 (69.2%) nursing staffs, 18 (12.4%) clinical officers, 14(9.6%) medical officers, while 13 (8.8%) comprise of other carders of health care providers according to the minimum criteria recommended. However, the facility has only one social worker and three data clerks. Summary is in Table 3 below:

This indicates that the health workers in the hospital are more experienced and have acquired adequate skills in their service delivery. This conforms to WHO (2003) guidelines that services providers are to be technically competent, experienced and motivated to provide services to adolescents as per their need/s.

The study found that for the facility to offers effective health services to adolescents, they is need to train staff on adolescent health however 143 (98%) of the staff indicated that they have never been trained specifically on adolescent health issues. Nonetheless, apart from the general training they received in college only 3 (2%) had received some training on adolescent health. Consequently in terms of the most useful topics for further training suggested by the healthcare providers, 127 (87%) stated that training on “substance use and misuse” would be very useful. 105 (72%) of all respondents stated that they felt it would be very useful for them to have training on “legal framework” issues (such as consent and confidentiality) and More than half 85(58%) considered training on “communication and consultation” with young people to be very useful. However, here were some notable variations by profession. 101(100%) of nursing staff stated that “legal framework” training would be very useful for them. Whilst this was the case for only 5 (35%) of doctors and 4 (33%) of general hospital health professionals. The health care provider identified learning need topics as indicated in Table 4 below;

**Table 4:** Learning needs as identified by health care providers

Topic	Response N (%)
Legal framework	105 (72)
Substance abuse	127 (87)
Communication and consultation with adolescents	85 (58)
Mental Health	26 (18)
Common medical problems and symptoms	34 (23)
Sexual and reproductive health	45 (31)
Chronic conditions and transition care	60 (41)

Healthcare providers felt that training needs to be flexible in order to take into account training already completed and to accommodate the different learning needs of staff with a range of knowledge and experience levels. WHO (2012) affirm that providers who are trained to work competently and sensitively with adolescents are often considered the single most important condition for establishing adolescent friendly services.

### 3.4 Skills

The study results have demonstrated that 124 (84.9%) healthcare providers communication with the patients is not proficient. However, 22 (15.1%) of the respondents reported that their communication is proficient enough for friendly health service delivery. Health care providers interviewed, 48 (33%) stated that not all staff were competent at various skills .However, 5 (60%) managers felt that some staff who had been in service a long time could do with training to refresh their skills in a number of areas such as how to communicate effectively with adolescents .Three (40%) managers believe that skills possessed by staff offering services to adult clients were largely transferable to child and adolescent services. However, it was also felt by 143 (98%) healthcare providers that it would be valuable for staff to have specific training on working with adolescents to improve communication skills. This is particularly useful during the transition to adulthood in adolescents with chronic illnesses .Therefore 110 (75%) of the healthcare providers felt that equipping the staff delivering these services with the necessary skills and competencies to deliver age appropriate care to adolescents is key to achieving service improvement. Lederman (2003) asserts that staff should have good interpersonal communication skills and be able to interact freely with young people, put them at ease, and encourage them to share their needs and concerns freely.

### 3.5 Knowledge

Majority 89 (61%) health care providers established that adolescent health services are not being effectively implemented. This is not surprising considering very few health have no clear knowledge on how adolescent health should be implemented. Almost all 130 (89%) health care providers who commented on this question expressed uncertainty about the extent of their knowledge. More than half 82(56%) stated that they may only work with an adolescent patient for a limited period of time and are therefore not able to fully observe or get to know an adolescent patient. Responses included awareness of some basic factors but not others such as social issues and mental health issues. 54 (37%) confirmed that they are not able to observe a young person’s behavior and would refer if there was a complicated issue rather than make decisions themselves. Almost two thirds 107 (73%) health care providers noted that they do not have the necessary knowledge. Several respondents 140 (96%) pointed out that they would like to receive training especially in communication. One of the health care providers emphasized this point as quoted that “*you have to communicate to them on their level” and “you have to make sure you don’t patronize them for them not to shy off”*. 142 (97%) of respondents agreed or strongly agreed with this statement. Godia (2010) asserts that training programmes need to be revised to ensure that staff are knowledgeable, skilled and welcoming, and that training is repeated and skills are updated.

### 3.6 Gender

More than half 99(68%) health care providers commented on this question and expressed the importance of behavior as a form of nonverbal communication for adolescents – One nurse was quoted that “*sometimes, they are trying to tell you something but at times they hold back if they are not comfortable with the gender of the health care provider especially if of opposite sex”*. On the other hand 151(71%) adolescents also reported that privacy and confidentiality are extremely important to them when making decisions about whether or not to seek reproductive health services because “*The female nurses always talk loudly and some of them like shouting therefore I am afraid that a they will share with a relative my sickness, I prefer a male doctor”*. However adolescents were generally forthcoming in speaking about sexual intercourse. Nonetheless 165 (78%) were exposed regarding the use of alternative expressions when talking about sexual activity in the presence of parents or strangers for example, “ *cleaning a gun* “ or “*putting Colgate on a toothbrush”* (Colgate refers to the semen and the toothbrush to the female genitals).

Kipchumba (2012) illustrated that some service providers do not have the required competencies to offer health services. Testing the relationship between health workers/staff’s competency and implementation of adolescent friendly health services, the study results indicated that there is a significant influence of the staff’s level of competence and experience in the service provision and the level of AFHS implementation in the hospital as established by (Ross & Elwood, 2008).

### 3.7 Findings from Focused Group Discussion

Qualitative data analysis revealed eight overarching themes across the four focus group discussion. Themes address both general clinical care, as well as friendliness of services

**Theme #1:** Positive Experiences with Care: Across the four focus groups, adolescents reported satisfaction with services and positive feelings about care. Adolescents discussed feeling listened to and understood by their health care providers, and many reported that they were “getting some of the things done.” A prominent sub-theme identified was that many respondents who reported satisfaction with care reported receiving services from same sex health care providers. There were also instances in which participants seen by health care providers of opposite sex also reported positive experiences with care.

**Theme #2:** Bridges and Barriers to Trust. In all focus groups, participants discussed a variety of factors that either adopted or inhibited the development of trust and a positive working relationship with providers. A lack of confidentiality was one such factor. Respondents raised concerns about information discussed being shared with other providers or being used against them. Also hindering the development of trust, several respondents discussed some providers being too harsh with instructions and this communicating that they do not really want to help them. Respondents in this focus groups noted that provider willingness to listen -even a little-would help to establish trust and communicate a willingness to be helpful. In turn, this would contribute to respondents opening up with providers and sharing more in care.

**Theme #3:** Stereotyping and Negative Experiences with Care. Across most focus groups, respondents reported a number of negative experiences with care in which they felt branded, insulted, or dismissed by providers. In addition to reports of feeling stereotyped and disrespected, respondents discussed a range of experiences in which they felt care was not useful, providers were disrespectful, their expressions of distress were misunderstood, they were not listened to, or they felt invisible to providers

**Theme #4:** Language Issues/Barriers. Participants discussed frustrations and difficulties they had participating in treatment when services were offered in English only. Further, many noted that not being able to communicate with providers contributed to feelings of isolation, loneliness, depression, anger, and, for many, resulted in their feeling as if they were being “ill-fated out of the system.”

**Theme #5:** System Challenges/Barriers to Care. System challenges and barriers to care were prominent themes that emerged in all focus groups. Respondents discussed a range of experiences that they felt obstructed their being able to receive effective care and address their needs. Examples of such experiences include, 1) Many clients being seen by only one health care provider, 2) waiting for long to be attended or being seen together with adults. Some even thought that private health facilities may give better services but they think that it may not be affordable to them.

**Theme #6:** Clinician and Agency Recommendations. In all focus groups, respondents offered a range of recommendations including: 1) Connecting with family and community; 2) Develop peer-based services and supports for adolescents ; 3) Develop additional services and supports for adolescents ; 4) Offer more education and training materials for adolescents; 5) Teachers should be empowered with the correct knowledge so that they can inform adolescents appropriately; 6) Parents should not leave all the work to the teachers, they should be buddies to their children and teach them freely. If they don’t do this most children will explore by themselves;7) Religious leaders to spread correct information to young people who nowadays are only a click away from so much inaccurate and even misleading data assert (Sawyer , Afifi , Bearinger, Blakemore, & Dick 012)

## 4. Assessing Health Resources

The link between health care resources and population health are not well understood. However, it is needless to argue that stock of assets and their composition, as inputs to the production of health, are important elements in the performance of health systems as results illustrate.

### 4.1 Physical Resources

Almost three quarters, 107 (73.3%) of the healthcare providers referred to the adolescent health services offered at the facility as friendly. Nonetheless, 39 (26.7%) indicated these services as unfriendly as there was no special room for adolescents to be attended.

Almost all 128 (88%) health care providers recorded their discomfort in offering certain specific services to adolescents such as reproductive health. Though according to 64 (44%) health care providers affirmed that the facility has adequate health resources to meet the adolescents’ needs.

Tilahun et al., (2010) supports this in their study in Ethiopia on health workers attitude toward sexual and reproductive health services for unmarried youth which expresses that some health workers were setting up penal rules and regulations against premarital sex.

### 4.2 Information Resources

According to 131 (89.7%) healthcare providers there are no relevant materials for them or for adolescents to read. Some 78 (37%) adolescents said that they prefer to learn about sensitive issues on their own, using written or audiovisual materials, because their discomfort level can be too great to retain information during a face-to-face session. Such material can be used while clients are waiting to be seen. Some materials should be available to take home for later review, particularly if the topics are complicated (such as symptoms of STDs). One adolescent suggested as quoted “Kuna *maugonjwa zingine tungependa kujua vile tunaweza zuia kama saratani au kuzuia mimba lakini tuna shindwa kuuliza daktari”* (There are some diseases like cancer or even how to prevent pregnancy but we are afraid to ask the doctor). This shows that the hospital is not fully equipped with information resources to offer quality services to the adolescents. The study by Farraley (2007) and supported by (Parker & Ratzan ,2010) assert this by highlighting that interactions and experiences of adolescents’ knowledge, attitudes and practices relevant in the health care system are framed through a health-literacy lens.

### 4.3 Medical Logistics

Both during needs assessment and the facility assessments, the basic equipment in the facility and laboratories were in working condition. However, the hospital is in need of specific adolescent equipment such as small sized specula to provide cervical cancer screening. The only serious shortcoming was that the supply of drugs and expendables was irregular, as reported by 117 (80%) health care providers. Unavailable right size condom have significant implications for adolescent friendly health services , given that increasing condom use is one of its key goals to help adolescents prevent unwanted pregnancy and STI/HIV. One doctor suggested that to effectively achieve availability of the needed supplies and services, the hospital should order supplies in good enough time, involve multiple actors in the supply chain management as well that the supply should be done by the government certified agencies (Sinclair, 2007).

In this study we can argue that resources are crucial components of health system. However, there is little systematic evidence on the impact of investments decisions on the performance of health. WHO (2007) emphasized that a well-functioning health system ensures equitable access to essential medical products, vaccines and technologies of assured quality, safety, efficacy and cost-effectiveness, and their scientifically Sound and cost-effective use

### 4.4 Health System Factors that can be put in Place to Support the Preparedness of Implementing Adolescent Friendly Health Services

To perform efficiently health systems require the combination of a large number of properly balanced physical and technical resource inputs. Policy makers must address a number of Questions as shown in the results below:

**Management Support**

Similar to the responses from health practitioners, all 8 (100%) managers highlighted the following areas as priority competency areas for training and development: legal and ethical frameworks around caring for young people; Communication and consultation skills with young people; Chronic conditions and transition care; And mental health in adolescents. Of these competency areas, 2 (25%) managers believe the most important areas for training and development were the legal and ethical framework with communication and consultation with adolescents. One manager working at outpatient setting identified substance use and misuse, weight and shape in adolescence as being other key areas for training and development.

When discussing the best formats for learning for their staff, all 8 (100%) managers felt that learning techniques were highly individual and a mixed method approach to delivery involving a mixture of job shadowing/on the job learning and e-learning would be the most appropriate format of delivery. However, it is interesting to note that other staff groups appear more frequently to think the identified areas are not very relevant to them, for example the hospital staff working with adults. This may be true in some instances but it may also be that some staff groups do not realize the relevance even though it may be relevant. This may also indicate an inappropriate reliance on others to take responsibility for aspects of adolescent care that staff find challenging. Esteves and Pastor (2000) confirmed that Sustained management support is related with “sustained management commitment”, both at top and middle levels during the implementation, in terms of their own involvement and the willingness to allocate valuable organizational resources.

**Health Workers Attitude**

Provider attitude was cited by 159 (75%) of adolescents’ as a barrier to accessing health services. More than half 82 (56%) providers confirmed this by reporting that they are often uncomfortable addressing the problems of adolescents. In addition, 50 (34%) providers had their own personal bias against providing adolescents with certain services like contraception or felt that adolescents should not be sexually active, thus hindering services to adolescents.

The adolescents illustrated that, the health workers rarely called the patients by their names as only 81 (38.2%) had ever been called by their names. However 16 (7.5%) indicated that the health workers sometimes called them by their names. Despite these sentiments, 177 (83.6%) adolescents reported that health workers listened carefully to them when visiting the hospital. 181 (85.3%) of the adolescents also indicated that, the health workers explained things to them in an understandable way.

Nevertheless 20 (9.4%) indicating that this was done sometimes but not always while 11 (5.2%) felt that the health workers did not explain things in an understandable manner. 84 (39.6%) of the respondents also felt that the health workers gave them sufficient time to ask questions regarding their health problems as 162 (76.4%) of them responded. According to 182 (85.8%) adolescents indicated that they liked the way they were served. On the other hand, 163 (76.9%) of adolescents indicated that they received service from health care providers who did not introduce themselves before attending them. Even though only 49 (23.1%) had visited the facility before. Table 5 below illustrates health workers attitude.

**Table 5:** Health workers attitude in Service Delivery to adolescents

	Yes	No	Sometimes
	n (%)	n (%)	n (%)
Staff introduced self	49 (23.1%)	149 (70.3%)	14 (6.6%)
Called Patient by Name	81(38.2%)	115 (54.3%)	16 (7.5%)
H/W Listened Carefully	177 (83.5%)	16 (7.5%)	19 (9.0%)
H/W explained things understandable	181(85.4%)	11 (5.2%)	20 (9.4%)
H/W granted opportunity for response	162 (76.4%)	34 (16%)	16 (7.5%)
Respondent treated to satisfaction	182 (85.8%)	17(8%)	13 (6.1%)
Respondent feels free with H/W	171 (80.7%)	30 (14.2%)	11 (5.2%)
Respondent felt confidential	171 (80.7%)	28 (13.2%)	13 (6.1%)
Had interrupted talks by other staff	71 (33.5%)	116 (54.7%)	25 (11.8%)
Sex of H/W an issue to respondent	43 (20.3%)	158 (74.5)	11 (5.2%)
H/W informed client about sickness	120 (56.6%)	84 (39.6%)	8(3.8%)

Hartline and Ferrel (1993), Sinclair, Crane, Hawton, and Williams, (2007) describe service quality as an attitude, perception or belief held about the manner in which service is delivered, which manner is itself reflected in the service encounters employees make with clients. Organizational culture and climate are both crucial characteristic of organizations that influence employees’ attitude (Aarons & Sawitzky, 2006; Carmazy & Aarons, 2003; Glisson & Hemmelgarn, 1998), and work attitude in particular predicts staff attitudes (i.e job satisfaction, organizational commitment) and subsequent staff turnover.

**Health Facility Organization**

116 (54.8%) adolescents indicated that they actually did not get the services. Those who sought but did not get the services were asked to state the reasons that made them miss the services. Majority 113 (53.3%) felt that the queues were too long. While those who had no money for services were 56 (26.4%). In addition, 27 (12.7%) found neighbors and felt ashamed. 133 (62.7%) adolescents interviewed at exit from the hospital said they desire not to mix with adults, and they would feel more comfortable if they were attended to in a separate room.

In the contrary 139 (95%) healthcare providers indicated that the waiting area was in good condition. Only 7 (5%) of the healthcare providers felt that the environment was not conducive for adolescents to stay while waiting for the services.

These results were in line with WHO (2004) guidelines on Provision of good quality health services to the adolescents through favorable policy environment and improved service provision.

The study also revealed similar results which indicated that most of the adolescents advocated for the following services to enhance implementation of friendly adolescents’ healthcare services; removal of service fee charged to seek health services 142 (67%), set up a separate room for adolescents 144 (67.9%), minimizing waiting time for adolescents 154 (72.6%), convenience in working hours 144 (67.9%), improvement on privacy 122 (57.5%) as well as provision of information materials for the adolescents to read while on the waiting bay which will create much knowledge on their understanding of adolescence. Also, 100 (47.2%) of the respondents felt that the staff at the hospital should be trained on offering friendly services to the adolescents. A summary of these results is provided in Table 7 below.

**Table 6:** Summary of adolescent responses on health facility organization

SERVICE AREA	n (%)
Long queues	113 (53.3%)
No money for service	56 (26.4%)
Found neighbors	27 (12.7 %)
No pleased with services	131 (61.8%)
Separate room	133 (62.7%)

**Table 7:** Suggestions by Adolescents to improve services

SERVICE AREA	Yes	No
	N (%)	N (%)
Eliminate service fee	142 (67%)	70(33%)
Set up separate room for adolescents’	144 (67.9%)	68 (32.1%)
Friendly staff	178 (83.9%)	34 (16.1%)
Shorter waiting time	154 (72.6%)	58 (27.4%)
Convenient working hours ( Evenings and weekend)	144 (67.9%)	68 (32.1%)
Improve on privacy	122 (57.5%)	90 (42.5%
Provide information material	133 (62.7%)	79 (37.3%)

Thus the findings show that Adolescents may find it difficult to obtain health services if the working hours coincide with times when they are busy with study, work or other activities this is according to Guidelines for Provision of Youth Friendly Services (2008).

## 5. Summary, Conclusion and Recommendations

### 5.1 Introduction

The broad objective of the study was to assess the preparedness of implementing adolescent friendly health services at Mama Lucy Kibaki Hospital. This chapter presents the summary, conclusion and recommendations based on the study objectives and the study results.

### 5.2 Summary of Findings

Age, sex, staff competence and level of education had significant positive influence on implementation of adolescent friendly health services while resource availability showed significant relationship between effectiveness and efficiency of service provision.

Health Resources corresponding to adolescent needs were information resources and infrastructure, having a separate room for consultation and having reading health information materials had significant relationship to implementation of adolescent friendly health services.

Health system factors significant were mainly infrastructure like management support and convenient working hours, waiting time and elimination of service fee which led the adolescents missing services.

### 5.3 Conclusions

The study found that staff capacity in preparedness for implementing adolescent friendly health services at Mama Lucy Kibaki Hospital was not adequate in terms of knowledge and skills in offering the following services; adolescent reproductive health, communication with adolescents, adolescent referral, counseling skills, and mental health substance abuse as well as the general adolescent health.

The hospital is not fully equipped with facilities to offer quality services to the adolescents. Health care providers need to have technical competence in working with adolescents in general, in the ‘adolescent-specific’ aspects of providing health promotion, preventive, curative and rehabilitative services, as well as in interpersonal relations and communication.

Availability of health resources on preparedness in implementing adolescent friendly health services was not sufficient. The hospital lacks relevant materials for the adolescents to read while waiting to see the health workers. However, despite this, there are plenty of supplies of relevant requirements of the hospital to meet the needs of the adolescents which include; condoms, oral pills, emergency contraception and injectable.

The hospital has limited (poor) communication systems regarding resource procurement, it lacks special sections for services provision, poor time management in the supply chain as well as poor supply chain management.

Special room for attending adolescents in the hospital, improvement in emergency management services, adolescent reproductive health program as well as the inclusion of the nutrition/obesity services.

The physical environment and procedures in the hospital are not always appealing to patients (adolescents).Negative response/attitude by some health workers, long waiting time, lack of private rooms for adolescents as well as health commodities specific to adolescents, and interruption by other health workers intruders while seeking medical advice from a health care provider are some of the factors affecting the friendlier services and would prefer seeking the hospital services again.

To enhance implementation of adolescent friendly health services; the hospital has to facilitate the removal of service charges to seek health services, set up a separate room for adolescents, minimize waiting time for adolescents, convenience in working hours, improvement of privacy as well as provision of information materials for the adolescents to read while on the waiting bay which create much knowledge on their understanding of adolescence.

### 5.4 Recommendations

This section presents some suggestions on how the problem under study could be solved. These are policy and implementation recommendations and areas for further study.

**Staff Capacity**

1) Need to train more service providers in dealing with adolescent so that they may be friendly and appealing to them, this will attract them to the clinics or facilities and in turn improve their health outcome.
2) There is need to train more school and college peer educators as well as teachers to compliment the health service providers in passing the adolescent friendly health service information. This will boost their capacity to offer comprehensive health services to adolescents.
3) Teachers and Parents should be Capacity built on issues of adolescent health.

**Staff Capacity**

4) Health facilities should use both human and physical resources within their reach to make their facilities more adolescent friendly. Where practicable, separate room should be allocated for servicing the adolescents within existing facilities.
5) Participatory adolescent information materials on health issues should be developed and be available at the service delivery points to complement services. Materials that can be taken away are also needed, so that adolescents can learn on their own. In addition, job aids that will assist providers in rendering services to the adolescents are also needed.
6) Without space (whether a room/ private corner or separate building), the service may never be adolescent friendly as many other issues revolve around this: Privacy, Confidentiality, Adolescent participation, Staff motivation, Stigma and Judgement.

**Health System Factors**

7) Health system within the facility should be strengthened to be more appealing to adolescents such as flexible consultation time. Consideration for adolescent consultation can be given for afternoons and weekends to enhance privacy.
8) Privacy and confidentiality should be increased through renovation of health facility, such as the partitioning of rooms or adding doors or screens. In addition, changes in provider practices, such as minimizing interruptions during client visits or shutting doors or windows when clients are being served, will also increase privacy.
9) In line with devolution, County Health Management Teams should consider inclusion of adolescent friendly health care in their plans of operations for sustainability. Monitoring systems and supportive supervision of AFHS delivery should be strengthened at the county level.
10) The government and partners should increase funding towards AFHS Implementation to enable service providers to offer these services completely free of charge to enable the school and college youth access them without any constraints

**Further Studies**

Similar studies need to be done in other county referral hospitals to generate more supportive evidence.

A comparative study between private and public hospitals to gauge the implementation of adolescent friendly health services to inform policy formulation and adjustment.

Studies should also be conducted to assess the barriers to implementation of adolescent friendly health services in both public and private health facilities.

It would also be important to find out if religion is associated with adoption of AFHS by health workers and what recommendations the government can get from the study findings.
